# The rise of congenital syphilis in Canada: threats and opportunities

**DOI:** 10.3389/fpubh.2024.1522698

**Published:** 2025-01-22

**Authors:** Ashorkor Tetteh, Victoria Moore

**Affiliations:** Centre for Communicable Diseases and Infection Control, Public Health Agency of Canada, Ottawa, ON, Canada

**Keywords:** congenital syphilis, vertical transmission, prenatal care, maternal syphilis, social determinants of health, SGBA Plus, barriers to health care, Canada

## Abstract

**Introduction:**

In Canada, rates of congenital syphilis have been increasing rapidly in recent years, following a surge in infectious syphilis. These trends call for a closer look at missed opportunities for testing, diagnosis, treatment, and follow-up of pregnant individuals. The epidemiological situation is especially serious given that effective treatment is available for syphilis during pregnancy and that congenital syphilis is a preventable outcome that engenders adverse birth outcomes such as miscarriage, stillbirth, and neonatal death as well as potentially lifelong ocular, neurological, hepatosplenic, and musculoskeletal sequelae. The objective of this study is to examine the factors associated with congenital syphilis trends and to highlight promising initiatives and programs across the country committed to addressing these trends.

**Methods:**

A literature review with a focus on Canadian studies was conducted to identify factors that may be driving the continued increase in early congenital syphilis rates over the past decade. An environmental scan of initiatives and programs providing syphilis care and support was also conducted.

**Results:**

Key factors identified in association with congenital syphilis outcomes included a lack of timely and repeated prenatal syphilis screening, inadequate prenatal treatment and follow-up of syphilis infection, barriers to accessing prenatal care caused by multiple intersecting social determinants of health as well as by certain structural determinants of health, and substance use. A number of initiatives to improve syphilis care within the health care system and several community-based programs filling in some of the gaps in syphilis care and support are making important advances in addressing the epidemiological situation with syphilis.

**Discussion:**

Much work is underway at various levels of government and local community to address the situation. Key recommendations for maximizing impact in curbing infectious and congenital syphilis rates include the following: planning an integrated strategy for addressing sexually transmitted and blood-borne infections as a whole; adopting a more holistic approach to improving health and wellbeing; developing targeted interventions for addressing structural and social barriers to health equity; and taking a collaborative approach to response by involving multilevel stakeholders, such as key populations, community groups, health care providers, and public health authorities.

## 1 Introduction

Rates of congenital syphilis have been increasing rapidly in Canada, particularly since 2018, following a surge in infectious syphilis in the country, with outbreaks reported in several provinces and territories (PTs) (1, 2). Infectious syphilis during pregnancy (henceforth referred to as prenatal syphilis) can have very serious consequences, including miscarriage or spontaneous abortion, premature birth, and stillbirth (3, 4). Congenital syphilis, which occurs when a baby is infected *in utero* (also known as vertical transmission) by the *Treponema pallidum* bacterium, can have significant and multisystem consequences for neonates, and untreated congenital syphilis can progress to late congenital syphilis and cause neurological and bone abnormalities (5). These consequences are preventable with timely testing and diagnosis followed by adequate treatment during pregnancy (3, 6). Yet the incidence of congenital syphilis in the country is increasing in spite of the existence of publicly funded health care services and of effective perinatal treatment (3, 7).

A better understanding of the reasons for this increase in congenital syphilis rates is highly warranted to inform policy, programming, and response. Closer attention needs to be paid to the reasons for the increasing incidence of prenatal syphilis and the missed opportunities among pregnant individuals for connection to prenatal care, timely testing and diagnosis, and adequate treatment.

The conceptual framework for syphilis infection in Canada, adopted by the Public Health Agency of Canada (PHAC) in 2020, proposes underlying structural and social determinants of health that influence proximate determinants experienced at an individual level, such as behavioral risk factors, violence, stigma and discrimination, and clinical factors, all of which can influence the risk of syphilis infection (8). This article will adopt the same framework in seeking to understand the reasons for the rising cases of congenital syphilis in Canada. We postulate that structural and social determinants of health, in particular, are largely responsible for the surge in cases of congenital syphilis. Drawing on the theory of intersectionality, we recognize that systemic factors often intersect to create overlapping and compounding inequities. This theory helps to explain why people whom systemic factors have already made vulnerable tend to be among the first to experience even more vulnerability in the event of disease outbreaks (9–13).

The aim of this study is to examine explanatory factors linked to rising congenital syphilis rates and to highlight the strength of downstream community-based responses through a few promising initiatives and programs across the country committed to addressing these trends.

## 2 Methods

### 2.1 Literature search strategy

National surveillance within a federated governance model is limited in its ability to monitor and parse sufficient risk factors, which are themselves inconsistently collected across jurisdictions, to help explain epidemiological trends. Therefore, a literature review focusing only on Canadian studies was conducted to investigate the structural and social determinants of health as well as clinical and individual risk factors influencing current trends in prenatal and early congenital syphilis rates. The conceptual framework guiding this investigation of explanatory factors in the Canadian literature defines “structural determinants of health” as factors that create unfair distributions of money, power, and resources. They include political, cultural, economic, and social structures as well as historical and ongoing colonialism and systemic racism. These structural factors also shape the conditions of daily life, which are the social determinants of health (14). Social determinants of health are the interrelated social, political, and economic circumstances in which people are born, grow up, live, work, and age. They have the potential to affect all aspects of the health of individuals, groups, and communities in different ways, and can intersect to shift and change conditions of daily life over time and across the life span (14). Together, structural and social determinants of health substantially influence the inequitable occurrence of perinatal and congenital syphilis across diverse populations (8). Health inequities are differences in health associated with structural and social disadvantages that are systemic, modifiable, avoidable, and unfair. These inequities put groups already experiencing disadvantage at further risk of poor health outcomes (14).

A research framework with predefined concepts, search strategies, and inclusion and exclusion criteria guided the search. The search was conducted by two independent reviewers, in collaboration with a PHAC librarian, through the Medline, Embase, and Scopus databases and was limited to peer-reviewed articles published from 2013 to 2023 in Canada. This timeframe was chosen to capture the 10-year timeframe preceding the execution of the literature search as well as to cast a wide-enough net to capture relevant changes in risk factors given that, in Canada, rates of infectious syphilis have been steadily increasing since the early 2000s, with the highest increases occurring after 2017 (2, 8). A gray literature search was also conducted using identical search strategies and was limited to only the first five pages of results. To minimize bias in the gray literature search, a short list of credible sources, including Google Scholar, CADTH Gray Matters, national, provincial, and territorial government websites, and public health association websites, was established prior to the search. Further details on the search and selection process, including inclusion and exclusion criteria, are provided in Supplementary Table S1 and Supplementary Figure S1.

## 3 Results

### 3.1 Why are rates increasing?

Based on the literature review, the most impactful factors influencing current trends in prenatal and congenital syphilis rates were revealed as a lack of timely and repeated prenatal syphilis screening, a lack of adequate treatment and follow-up of syphilis infection during pregnancy, barriers to accessing health care, including prenatal care, caused by multiple intersecting social determinants of health as well as by certain structural determinants of health, and substance use.

#### 3.1.1 Lack of access to appropriate syphilis screening

Timely and repeated syphilis screening and diagnosis during prenatal care increases the likelihood of detecting infections as early as possible in pregnancy as well as reinfections occurring later in pregnancy following adequate treatment of initial infections. It therefore plays a critical role in the prevention of congenital syphilis (15, 16). Moreover, numerous studies have shown that having access to prenatal care in and of itself does not guarantee timely and repeated prenatal screening for syphilis (17–21).

The timing of the first prenatal syphilis screening test has been found to be strongly associated with a congenital syphilis outcome. For example, one study found the odds of giving birth to an infant with congenital syphilis to be eight times higher among birthing parents first screened in the third trimester (AOR 8.4, 95% CI 2.9–24.6) or in the last month of pregnancy (AOR 8.1, 95% CI 1.4–47.8) compared with those screened in the first or second trimesters (20). However, although national, provincial, and territorial screening guidelines all recommend universal screening of pregnant individuals in the first trimester or at the first prenatal visit, several publications from Alberta, Quebec, and Manitoba have reported cases of congenital syphilis resulting from birthing parents receiving their first screening test for syphilis late in pregnancy (18–21).

Several Canadian and international researchers have highlighted the limitations of using a risk-based strategy to identify individuals who require repeat screening, in the current epidemiological context. Firstly, the stigmatization associated with risk factor identification or low awareness of congenital syphilis prevention may lead to a lack of disclosure by patients/clients (22, 23). Secondly, this approach requires providers to be knowledgeable about when the risk is high (22), however limited awareness of and education on existing guidelines among prenatal care providers has been reported (20, 24, 25). Thirdly, health care providers may not sufficiently screen individuals for risk factors due to discomfort in discussing sexual health or a lack of capacity, among other factors (26, 27).

A lack of repeated prenatal screening for syphilis appears to be implicated in the rising rates of congenital syphilis. Despite National Advisory Committee on sexually transmitted and blood-borne infection (NAC-STBBI) guidelines, developed in collaboration with PHAC, recommending repeated screening at 28–32 weeks of pregnancy (during the third trimester) and again at delivery in areas with syphilis outbreaks or for pregnant individuals facing ongoing risk of infection or reinfection (28), and despite several jurisdictions revising their guidelines in response to ongoing outbreaks to recommend universal repeated prenatal screening for syphilis during the third trimester and/or at delivery (Alberta in 2009; Manitoba in 2019; British Columbia in 2019; and Québec in progress for 2024) (17, 29–31), many pregnant individuals are still not retested for syphilis. Numerous studies have documented cases of congenital syphilis occurring in pregnancies that initially screened negative or were suspected of reinfection, where rescreening was provided only close to delivery or after delivery (17–21, 32–34). Where repeated screening has been implemented as recommended, however, greater proportions of pregnant individuals have been rescreened during pregnancy (29, 30) and additional cases of prenatal syphilis have been captured, including in instances of no previous screening or previous negative screening tests (17).

#### 3.1.2 Lack of adequate syphilis treatment and follow-up during pregnancy

Timely treatment of prenatal syphilis, both at initial diagnosis and at reinfection, is critical in preventing congenital syphilis. Several studies have demonstrated the very strong association between treatment of prenatal syphilis and a congenital syphilis outcome (20, 35, 36). Specifically, factors such as not receiving any treatment at all, initiating treatment too close to delivery, and not completing the full treatment course have resulted in cases of congenital syphilis (18, 32, 33, 37), sometimes in spite of a prenatal syphilis diagnosis earlier in pregnancy (21, 38).

Furthermore, several studies have proven that access to prenatal care does not guarantee adequate treatment of prenatal syphilis (20, 21, 33, 38, 39). This reinforces the need to complement appropriate clinical screening guidelines for pregnant individuals with timely and adequate treatment of cases.

The delay between a positive screening test or a diagnosis of syphilis and the initiation of treatment is an issue of particular concern among pregnant individuals who do receive prenatal care and syphilis screening tests. In one study, it was found that 25% of the pregnant individuals experienced a delay of more than 48 days between their screening test and the commencement of their treatment. In this study, analytical results showed that individuals with infectious syphilis who birthed an infant diagnosed with congenital syphilis received their treatment later in pregnancy, compared to individuals with infectious syphilis birthing infants who were not diagnosed with congenital syphilis (AOR 1.04, 95% CI 1.02–1.06), and that after the end of the 32nd week of pregnancy, there is a high likelihood of a congenital syphilis outcome, even if treatment is administered (19).

#### 3.1.3 Barriers to accessing health care/prenatal care due to social determinants of health

Having access to syphilis testing and treatment first requires having access to health care, or in the case of pregnant individuals, prenatal care, and herein lies one of the great barriers for individuals experiencing health inequity. In Canada, financial barriers to health care services are mitigated by publicly funded health care systems that cover aspects of prenatal medical care for most residents (e.g., physician visits, hospital stays, diagnostic tests, etc.) (40). Thus, it is rather factors such as immigration, geography, sex, age, income, and housing that have been shown to play an important role in the disproportionate impact of syphilis on some individuals and communities. Race and ethnicity data are not collected systematically or in a standardized manner among Canadian studies, and only a few studies have investigated the association between race/ethnicity and congenital syphilis (18–20, 36, 39, 41). Moreover, reliance on self-identification for race/ethnicity data in these studies presents several limitations. Reporting bias is one such limitation, whereby individuals may be reticent to self-identify due to fears of stigma and discrimination, particularly in settings where data collection is not culturally safe and appropriate. For these reasons, this article does not examine race/ethnicity as an independent social determinant of health, but rather focuses on the structural and social determinants of health interacting with and shaping race/ethnicity-based health inequities.

Specifically in Quebec, several studies have shown that birthing parents who reported migrating to Canada shortly before birth or having a partner living outside Canada represented the majority of inadequately treated prenatal syphilis cases (42) and were commonly implicated in congenital syphilis outcomes (18, 33, 43). Studies have highlighted factors such as difficulty accessing health care, low awareness of free prenatal classes, challenges in navigating the Canadian health care system, culturally inappropriate care (44), limited language proficiency, and overshadowing priorities in integration, such as finding housing, employment, and food, as playing a role in immigrants receiving inadequate prenatal care (45). Notably, certain groups living in conditions of vulnerability, such as citizens who are underhoused or experiencing homelessness and have lost or never obtained health cards, temporary residents with expired work or education permits, and undocumented migrants, do not have access to Canada's publicly funded health care insurance coverage (7, 46).

One's geographic location also greatly impacts both one's exposure to syphilis infection and one's access to prenatal care. Studies in Alberta, Quebec, and Manitoba have discussed how the spread of syphilis infections to rural and remote areas, where early access to health care services may be restricted, could contribute to the increase of congenital syphilis in these locations (18, 20, 47). For example, in Quebec, the northern region of Nunavik has been the area of the province most impacted by congenital syphilis between 2016 and 2021, with a disproportionately high rate of infectious syphilis of 477 per 100,000 population in 2020 (compared to a rate of 31.4 per 100,000 population in Montreal, the region with the second-highest rate in the province, in the same year) (18). Furthermore, there are very limited studies providing insight on the role of mobility within or between PTs in the incidence of infectious syphilis in pregnant individuals leading to a higher occurrence of congenital syphilis. Among three cases of congenital syphilis reported in Quebec in 2020 (one probable early congenital syphilis, one confirmed early congenital syphilis, and one confirmed syphilitic stillbirth), one involved a birthing parent who had traveled from a northern region outside of Quebec and was assessed in Quebec late in pregnancy (18).

In terms of sex as a social determinant of health, national epidemiology has shown that rates of infectious syphilis are rising the fastest among females (2), and indeed in Quebec geography and sex intersect to create a condition where females in Nunavik experience the highest rates of infectious syphilis in the province (694 per 100,000 females in 2020), relative to males (18). Nationally, the PTs with the highest rates of infectious syphilis are also the PTs experiencing the highest proportions of infectious syphilis among females relative to males and, inevitably, the highest rates of congenital syphilis (1, 48). Some studies have indicated that anatomical differences in females may make primary lesions take longer to identify because they are internal, which may play a role in females seeking health care and syphilis testing later in their infection (49, 50). Other factors related to women's social position in society may also play a role in delayed health care–seeking behavior (47).

Geography and sex further interact with age in the national epidemiology of infectious syphilis, which demonstrates that females living in the PTs most impacted by infectious syphilis experience the highest rates in the 20-to-29-year age bracket (48). In other words, young females in their twenties living in the most impacted PTs are the most at risk of acquiring a syphilis infection and represent a key population requiring high attention to both protect the health of pregnant individuals and combat the high rates of congenital syphilis in Canada. Numerous studies corroborate the finding that this age group, in particular, among females experiences vulnerability. Preliminary results from a Canadian Pediatric Surveillance Program (CPSP) study co-led with PHAC indicate that the median age of birthing parents is 27 years (range: 17–39 years) (38). Similarly, studies in various PTs have reported similar median ages of birthing parents of congenital syphilis cases, including Manitoba (26.5–27.0 years), Alberta (26.0–27.5 years), and Quebec (20–29 years) (19, 32, 51).

Income insecurity, or poverty, is widely recognized as a common, underlying factor intersecting with and influencing homelessness and unstable housing. Studies have shown income insecurity to be linked to a higher observed frequency of prenatal syphilis (17, 19, 32), though not statistically associated with a congenital syphilis outcome (19). Similarly, homelessness and unstable housing have been linked to a higher observed frequency of prenatal syphilis (8, 17), but this link has not been statistically associated with a congenital syphilis outcome (20).

#### 3.1.4 Barriers to accessing health care/prenatal care due to structural determinants of health

Gaining access to health care in order to benefit from syphilis testing and treatment is also impeded by structural determinants for many communities. The structural barriers are multiple and include a lack of transportation, a lack of paid time off for medical appointments, language barriers, fear of stigmatization, fear of child apprehension, lack of access to government childcare services, fear of being reported to immigration authorities, newcomers' challenges accessing and navigating the Canadian health care system, lack of health insurance for people experiencing vulnerability, and challenges with the organization of health care across Canada that make it difficult to access primary health care providers or timely health care services (7, 19, 32, 44, 46, 52–54). Furthermore, experiences of stigmatization and discrimination within the health care system, rooted in Canada's history of colonization and maintained by ongoing colonialism, systemic racism, and discrimination toward gender-diverse individuals have contributed to fear and mistrust of and reluctance to use mainstream health care services for many communities (8, 55, 56), potentially contributing to missed or late syphilis testing. In the context of diagnosing syphilis among pregnant individuals, studies have also suggested a strong link between fear of child apprehension and disengagement from, avoidance of, or delayed presentation to prenatal care services (19, 57–62). Pregnant individuals who are experiencing factors that might put them at risk for separation from their infants, such as a lack of secure housing, poverty, or drug use, may avoid interacting with the health care system so as not to be reported to the child welfare system. However, inadequate prenatal care also often leads to the perception that the individual is an unfit parent, further increasing the risk of child apprehension at birth (59). As such, many social determinants of health play into and are impacted by structural determinants of health, which collectively help explain the delays and missed opportunities for pregnant individuals in accessing prenatal care in general and syphilis testing in particular.

#### 3.1.5 Substance use

Substance use has been widely linked across multiple studies to delays in accessing prenatal care (19, 41), prenatal syphilis outcomes (17, 20, 43), inadequate treatment of prenatal syphilis (38, 45, 62, 63), and congenital syphilis outcomes (18, 32, 36). The use of methamphetamine and injection drugs, especially, is generally linked with having multiple sexual partners or concurrent sexual partnerships, inconsistent condom use, and the exchange of sex for drugs or money, which are all behaviors that elevate the risk of acquiring syphilis and other STBBIs (64, 65). Additionally, people who use drugs are more likely to experience stigma and to express mistrust of the health care system (64). In particular, as previously mentioned, pregnant individuals who use substances may be reluctant to use mainstream health care services due to fears of the involvement of the child welfare system (59). Substance use is a proximate, or individual-level, determinant of health that is impacted by wider social determinants of health. Thus, substance use, coupled with social determinants of health such as unstable housing and poverty, and structural determinants such as systemic discrimination and child apprehension policies, may contribute to reduced health care system use and increased risk of syphilis infection in pregnant individuals (19, 55, 66).

The Prairie provinces, which observed the highest rates of congenital syphilis in Canada in 2022 (1), have been experiencing a concurrent substantial increase in the availability of and harms associated with methamphetamine use in the last few years (67).

### 3.2 Key findings

In summary, the reasons for the increase in rates of prenatal and congenital syphilis are multifold. Proximate determinants of health experienced at an individual level, particularly substance use, may increase the occurrence of higher-risk sexual encounters and behaviors and the neglect of safer sex practices such as condom use (66). Numerous intersecting social and structural determinants of health, examples of which have been discussed in detail above, prevent many people with infectious syphilis, including those who are or later become pregnant, from accessing the health care they need to get appropriately tested, treated, and followed up clinically (8). Consequently, rates of infectious syphilis among females in their reproductive years have been increasing rapidly (2). In addition to these factors, pregnancy-specific considerations, such as the mistrust and avoidance of the health care system during pregnancy due to fears of stigma or child apprehension (59, 66), contribute to rising rates of congenital syphilis. Furthermore, when prenatal syphilis screening does not occur in a timely manner, preventing the early detection of infection, fetuses are exposed to *treponema pallidum* bacteria *in utero* for a longer duration and are thus at higher risk of acquiring congenital syphilis (4). When prenatal syphilis screening is not repeated, infections that were too new to be detected and infections that were acquired after the initial negative test result are left to progress (32). Receiving no, partial, or late (i.e., < 4 weeks before delivery) treatment and follow-up of syphilis infection during pregnancy results in the prenatal syphilis infection continuing to pose a risk for vertical transmission during pregnancy or childbirth (19). All of these different factors combine to create the perfect storm for the surge in congenital syphilis rates, and they will collectively need to be addressed to find sustainable solutions for the prevention and control of congenital syphilis in Canada.

### 3.3 Opportunities and ongoing initiatives to address the rising rates of congenital syphilis

The persistence of structural, social, and individual barriers to preventing prenatal and congenital syphilis points to opportunities to improve health systems and the way in which health care is delivered, particularly to populations experiencing social and health inequities, as well as opportunities to financially support promising initiatives led by community-based organizations. An analysis of these opportunities was conducted in addition to the literature review to highlight the strengths of community-based initiatives. Such downstream initiatives are often grounded in addressing systemic barriers, such as stigma, discrimination, and broader health inequities. Upstream opportunities for overcoming systemic barriers have already been discussed elsewhere (48). The analysis that follows sheds light on a few initiatives underway across the country that are seizing some of these opportunities to combat the spread of syphilis infection.

#### 3.3.1 Health care system opportunities

Addressing the missed opportunities in prenatal care for preventing the vertical transmission of syphilis is crucial for reducing congenital syphilis rates across Canada (6). Several strategies can be adopted to bridge this gap, including taking a closer look at point-of-care test (POCT) deployment to appropriate locations and considering alternative settings and models of care.

##### 3.3.1.1 POCT deployment

Pai and colleagues define point-of-care testing as diagnostic testing that will result in a clear and actionable management decision such as when to start treatment or to require a confirmatory test, within the same clinical encounter (68). POCTs can be a valuable additional tool in preventing congenital syphilis given their ability to deliver rapid results and enable immediate empiric treatment in a single visit. POCTs are particularly useful in situations where timely intervention is necessary, such as during labor or delivery for birthing parents who have received limited or no prenatal care, in settings with limited laboratory capacity, in situations involving hard-to-reach populations or where barriers to follow-up exist, and in settings where the pre-test probability of syphilis infection is high, such as in communities experiencing outbreaks (bearing in mind the limitations of POCTs, including that treponemal tests cannot distinguish active from prior syphilis infections and that combination treponemal/non-treponemal tests are preferred during sustained outbreaks) (69).

Multiple research efforts are ongoing to integrate POCTs into clinical and public health practice in Canada. In March 2023, MedMira, a biotechnology company specializing in diagnostic testing, began clinical trials for its Reveal Rapid™ TP (Syphilis) antibody test in Saskatchewan and British Columbia (70). In parallel, the STAR Study evaluated the field accuracy of the Chembio DPP^®^ Syphilis Screen & Confirm as a rapid diagnostic test in isolated Canadian Arctic communities (71). The STAR Study's results suggest that integrating rapid diagnostic tests like the Chembio DPP^®^ POCT into broader syphilis testing strategies in remote Northern regions could substantially reduce the time between testing and treatment, thereby preventing complications of syphilis in affected individuals, limiting onward transmissions, facilitating timely treatment, and minimizing the risk of loss to follow-up (69).

Clinical data from the Point-of-Care Tests for Syphilis and HIV (PoSH) Study, which evaluated the performance and acceptability of two dual human immunodeficiency virus (HIV)/syphilis POCTs (the bioLytical INSTI^®^ Multiplex HIV-1/2 Syphilis antibody test and the MedMira Multiplo Rapid TP/HIV test) in outreach and acute care settings in Alberta, supported the March 24, 2023, Health Canada authorization of the bioLytical INSTI^®^ Multiplex POCT (72). This is the first and only syphilis POCT currently approved for use in Canada to date (73). However, this test has several limitations including, notably, its authorization for use only in clinical or laboratory settings by trained personnel and in patients with signs and symptoms of syphilis and HIV (73). Confirmatory serological testing is still required to distinguish between an active or past infection when a POCT test result is positive, to support the assessment and diagnosis of congenital syphilis, to confirm adequate response to treatment, and to diagnose re-infections (74).

Despite these limitations, POCTs can improve the quality of health care for individuals experiencing structural barriers, such as rural or remote residence or health care–related stigma and discrimination, by increasing access to testing, reducing treatment delays, and ensuring continuity of care (52). Furthermore, a recent study synthesizing literature on the cost-effectiveness of syphilis screening in pregnant individuals provided evidence that rapid testing, in combination with high treatment rates, is generally the most cost-effective strategy for screening pregnant individuals and subsequently for preventing congenital syphilis (75). However, the optimal use of POCT will vary based on setting and population, due to factors such as adequate testing infrastructure and capacity and access to confirmatory testing, historical serological test results, clinical assessment by a licensed health professional, and treatment, highlighting the importance of having flexible settings and models of care (76).

##### 3.3.1.2 Alternative settings and models of care

Expanding syphilis screening beyond traditional care settings can help reach individuals who may not otherwise have equitable access to prenatal care (6). Moreover, providing screening in alternative settings rather than physician offices may foster a more comfortable and anonymous environment for disclosing and treating STBBIs from both patient/client and provider perspectives (27). Alternative settings and models of care may include community health centers, mobile and pop-up clinics, pharmacies, emergency rooms, and harm reduction and peer outreach programs.

Mobile clinics, by bringing health care services into areas of high need, rather than having individuals travel to seek out these services, can help improve access to syphilis screening for populations who face barriers to accessing screening in traditional settings. For example, the Wellness Wheel Clinic, a community-led health care program, works through local health centers to maintain an urban clinic in Regina, Saskatchewan, while sending outreach medical teams on regular visits to rural and remote Indigenous communities, including on-reserve First Nations communities, to provide health care, including STBBI testing (77).

Pop-up clinics can also be an effective way to test individuals in outbreak areas. For example, in response to the rising number of syphilis cases, local community clinics and health care providers, in partnership with the Government of the Northwest Territories, hosted two pop-up clinics in Yellowknife, brought rapid HIV and syphilis testing to communities, and installed more than 300 free condom dispensers in public locations in all communities in the territory (78).

Pharmacy-based models of care provide an opportunity to integrate innovative testing and treatment approaches into existing community health infrastructure. Pharmacies can reach individuals who might not seek testing in traditional settings, either due to stigma or geographic isolation. Through partnerships with public health authorities, health care providers, and local community organizations, pharmacies can acquire test kits, provide educational materials, and facilitate confirmatory testing and linkage to care (79). For example, as part of an ongoing implementation study, select community pharmacies in Newfoundland and Labrador, Nova Scotia, and Alberta use POCTs to offer free HIV, syphilis, and hepatitis C screening and ensure individuals receive the necessary follow-up care and support through pre-established partnerships and referral systems (79, 80).

Emergency rooms also provide a key opportunity for syphilis screening interventions. In 2021, Alberta Health Services introduced a Best Practice Advisory (BPA) within Connect Care, its central clinical information system, that can prompt emergency department providers to order a syphilis test for pregnant individuals without a history of prenatal screening as well as can initiate Partner Notification Nurse involvement for timely outreach and treatment in the event of a positive test result (81). Partner screening, despite persistent barriers like stigma, fear of testing positive, and limited awareness of screening risks and benefits, is essential in preventing congenital syphilis as it disrupts transmission and reduces the chance of reinfection (82). Evaluation of the syphilis BPA's effectiveness in reducing infectious and congenital syphilis across Alberta is ongoing (81).

#### 3.3.2 Opportunities to support initiatives led by community-based organizations

The initiatives described above illustrate effective community-based approaches to improve syphilis screening and treatment efforts. These initiatives highlight the importance of community partnerships and targeted outreach strategies for enhancing syphilis testing and treatment in nontraditional and alternative care settings.

Furthermore, providing person-centered wraparound care to populations experiencing intersecting determinants of health is critical to reducing cases of infectious syphilis and consequently congenital syphilis. A number of community-based organizations have adopted this wraparound approach to health care service delivery.

The Healthy, Empowered, Resilient (H.E.R.) Pregnancy Program at Boyle Street Community Services in Edmonton, Alberta, is a community-based intervention that helps street-involved pregnant individuals access health care and social resources. Using a harm reduction approach, the program empowers these individuals to meet their pregnancy goals, offering health education, system navigation services and referrals, prenatal care, STBBI testing, and pregnancy drop-in support (83). An evaluation of the program spanning from April 2011 to July 2013 demonstrated the program's success in supporting pregnant individuals in feeling empowered, facilitating their access to social and health resources, and helping them achieve their health and parenting goals (83).

The Pregnancy Pathways program, also based in Edmonton, Alberta, provides prenatal care and social support services to pregnant individuals experiencing homelessness or facing precarious housing (84). In collaboration with 25 community partners across acute health care, addictions, mental health, government, and non-profit sectors, the program aims to stabilize pregnant individuals experiencing homelessness by providing them with their own apartment, a space to build community and foster peer support, and access to wellness coordinators who work with clients to build life and parenting skills (85).

Call Auntie, a similar community-based outreach program based in Toronto, Ontario, provides wraparound support, including mental health services, primary care, specialist referrals, and social support programs, all grounded in Indigenous Ways of Knowing and Being (86). Call Auntie also runs weekly clinics at the Toronto Birth Centre and collaborates with partners to offer sexually transmitted infection (STI) testing, treatment plans, and additional support with accessing food, traditional medicines, and other community resources (86).

Community-based interventions such as those discussed above, prioritize culturally sensitive and trauma-informed care to promote access to and retention in prenatal care. These initiatives provide a range of comprehensive support services tailored to the unique needs of pregnant individuals, empowering them to make informed decisions about their health and contributing to better health outcomes for both parents and newborns.

## 4 Discussion

We have reviewed key factors that can help explain the rise of congenital syphilis rates in Canada. We have also reviewed opportunities to address the rising rates through different approaches to care and program delivery both in the health care system and within communities.

The results of the literature review unveil the important role of structural, social, and proximate determinants of health in shaping prenatal and congenital syphilis outcomes in addition to other comorbidities experienced by pregnant individuals (55). In this syndemic health context, whereby multiple health problems co-occur, interact, and combine to exacerbate the health burden among populations experiencing vulnerability (87), syphilis emerges as just one symptom out of many other health and social needs that require attention in the lives of pregnant individuals.

The analysis of initiatives led by community-based organizations demonstrates opportunities to address these problems through person-centered approaches that consider the competing health and social needs of the person seeking care. In addition, the examination of health care system opportunities illustrates the value of offering services, such as POCTs or mobile clinics, that are tailored to the specific needs of a community. The flexibility of the health care system to adapt and deliver services in this manner fosters a more inclusive and responsive approach.

Addressing current trends in congenital syphilis in an effective manner will require both a deep understanding of gaps in prevention and care and the development and support of effective strategies to address them and to meet national and global commitments to congenital syphilis prevention and control.

### 4.1 Implications

Canada has committed to the World Health Organization's 2030 global targets for lowering rates of infectious and congenital syphilis. These targets aim for 80% of countries to have 200 or fewer cases of confirmed and probable congenital syphilis live births and stillbirths per 100,000 live births per year by 2025, and 50 or fewer cases of confirmed and probable congenital syphilis live births and stillbirths per 100,000 live births per year by 2030 (88). The Pan-Canadian STBBI framework for action and Government of Canada's STBBI Action Plan 2024–2030, reiterate this commitment. The Action Plan outlines the federal strategy for moving toward these goals and other STBBI targets (89, 90). It is expected, from the health care gaps identified in the literature review and the opportunities for addressing these gaps revealed in the review of promising initiatives and programs, that this alignment of global and national goals will help unroot underlying health inequities and achieve better outcomes for congenital syphilis.

The literature review supports the need for different approaches to the prevention and control of congenital syphilis in Canada. These approaches must go beyond merely connecting individuals to prenatal care and syphilis testing. They must involve an intersectional and collaborative strategy and leverage innovative models of health care service delivery that are adapted to local contexts and that offer packages of health care and social service support to patients rather than single-disease-focused care. Employing a sex- and gender-based analysis plus (SGBA Plus) framework is important in this path forward. SGBA Plus is an intersectional approach to assess how factors such as sex, gender, age, race/ethnicity, socioeconomic status, disability, sexual orientation, cultural background, migration status, and geographic location interact and intersect with each other and broader systems of power (91). Applying this lens to future studies and programs will help researchers, analysts, and program staff to better expose gaps or opportunities to improve equity and inclusion in health care programming, access, and service delivery in Canada.

Building on this, the review of promising initiatives and programs has shown that different approaches, including alternative models for delivering care, together with expanded partnerships and collaborations between federal, PT, local, and community leaders, are critical. They will help to better align upstream policy, programming, and funding with downstream grassroots leadership and efforts to achieve the common goal of fostering healthier communities and, ultimately, reducing congenital syphilis rates.

Various sectors have an important role to play in achieving this common goal. For health professionals, offering comprehensive, culturally safe and stigma- and discrimination-free STBBI and sexual health care can empower patients to seek timely testing and treatment (89, 90). Policymakers could focus on reviewing laws and policies that affect access to health care and essential services and prioritize investments in programs that address the broader determinants of health (89). Additionally, community organizations play a vital role in providing targeted and localized interventions, raising awareness, and facilitating access to care for key populations (90).

Strategic funding also has an important role to play in this intersectoral work. Building progressive public health systems requires integrated funding approaches that support diverse communities and emphasize holistic strategies over disease-focused models. Investments in programs and services like harm reduction or supportive housing not only mitigate immediate risks but also contribute to improved prenatal health outcomes, as exemplified by the community-based intervention discussed above.

### 4.2 Syphilis infection disproportionately affects those in situations of greatest poverty and vulnerability

Improving overall living conditions and access to respectful, patient-centered prenatal care could have an important impact on the reduction of congenital syphilis (8, 55). Public health responses to rising syphilis rates should carefully consider how to support pregnant individuals in prioritizing their health care needs in a context where competing, fundamental needs such as housing, food, and a living wage are not met. Ensuring health services are provided in a culturally safe and trauma-informed manner could furthermore create the appropriate enabling environment to encourage pregnant individuals who fear stigma, discrimination, and the apprehension of their newborns to seek prenatal care and testing and prevent congenital syphilis (8, 90, 92–94). In this manner, the disproportionate impact of syphilis on those living in situations of greatest poverty and vulnerability may be alleviated.

### 4.3 Limitations

Not only are studies examining the rise in syphilis rates at the national level in Canada rare, but there are also limited PT-specific or region-specific studies in Canadian literature investigating the structural, social, and proximate determinants of health and other risk factors influencing the current epidemiology. As a result, regional disparities could not be examined and the findings on explanatory factors in a limited number of PTs is also not generalizable to all regions of the country. There is a need for studies in other PTs or through interprovincial/territorial collaborations examining explanatory factors for current rates of congenital syphilis across the breadth of the country. More national studies are also needed to enable better generalizability of study results to the Canadian population. Furthermore, more scientifically rigorous studies are needed to properly evaluate statistically significant associations, rather than frequency distributions, between congenital syphilis and its risk factors. Not all relevant studies may have been published, due to publication bias, or may have been identified in the literature search, despite the systematic approach to conducting a thorough and comprehensive search. Among the studies reviewed, it is possible that some relevant studies were missed in the screening process or that some of those screened in have methodological biases that may limit the ability to generalize their results to the wider population.

Additionally, while this article highlights some opportunities and ongoing initiatives to address rising rates of congenital syphilis, it should be noted that some of the initiatives described may lack sufficient evaluation metrics to demonstrate their overall effectiveness in reducing congenital syphilis rates. It must also be acknowledged that many more community-based organizations across Canada are conducting valuable work on STBBIs, and specifically, syphilis prevention, and due to space constraints and the scope of this article, not all of these organizations or their efforts could be described. Moreover, there may be ongoing work by community-based organizations that has not yet been published and, therefore, could not be included in this analysis.

## 5 Conclusion

The increase in rates of congenital syphilis in Canada appears to be linked to prenatal care factors such as late or no interactions with the prenatal care system, inadequate, late, or no syphilis testing, late or no syphilis treatment, and gaps in the follow up of patients after testing or treatment, in addition to multiple intersecting structural, social, and proximate determinants of health. Research incorporating more quantitative methods are needed to validate the primarily qualitative findings of the studies included in this article. More PT, regional, and national studies examining explanatory factors for the current rate trends are also greatly needed to enable a better understanding and generalizability of these factors across the Canadian population. Having more standardized data collection instruments and more detailed data collection on risk factors will greatly help to meet this need at the level of PT and federal public health surveillance. Longitudinal studies in PTs where congenital syphilis rates are increasing are also urgently needed to identify gaps and to inform policy and programming for rapid and targeted congenital syphilis responses. This article is focused on the Canadian context, and its findings are therefore not generalizable to international contexts. Given the increasing global concern around congenital syphilis, we suggest future research examine international initiatives, such as Brazil's Syphilis No! Project (95), a major national syphilis response initiative in that country, to advance global efforts in addressing this urgent issue. Future research could also consider a more in-depth examination of other key determinants of health such as immigration, socioeconomic status, and access to health care services like primary care, to better understand their influence on prenatal care outcomes.

Preventing congenital syphilis will require a multilevel, intersectional, and collaborative approach that addresses structural and social barriers to health equity. Collaborating with communities, especially key populations, in the development and implementation of syphilis interventions will ensure cultural relevance and the tailoring of approaches to local contexts. Coordinated response efforts by federal, provincial, territorial, and Indigenous partners, along with health care providers and local communities, are urgently needed to maximize response impact and reduce congenital syphilis rates across jurisdictions. Public health authorities can leverage partnerships with prenatal and women's health groups to increase awareness of the risk of vertical transmission and available prevention programs, as well as to increase funding for STBBI services (96). Furthermore, health and social service providers can play a significant role in educating pregnant individuals about syphilis and supporting access to prenatal care, including syphilis testing and treatment, as well as to wraparound services (90, 97).

Reducing the rising rates of congenital syphilis in Canada demands immediate action and will require targeted interventions tailored to the needs of affected populations. An integrated approach to addressing STBBIs collectively is critical, given the shared risk factors, behaviors for transmission, and common transmission routes among STBBIs (8, 89). Aligned with the Government of Canada's STBBI Action Plan 2024–2030, an integrated STBBI strategy not only maximizes impact in curbing infectious and congenital syphilis rates but also makes strides in addressing other STBBIs that often impact the same populations, promoting a more holistic approach to improving the health and wellbeing of individuals (90).

## Supplementary material

Supplementary Table S1 Literature search inclusion and exclusion criteria.

Supplementary Figure S1 Flow chart of the literature search and selection process.

Supplementary French abstract S1 La hausse de la syphilis congénitale au Canada: menaces et opportunités.

## Glossary

bioLytical INSTI^®^ Multiplex HIV-1/2 Syphilis antibody test: a rapid diagnostic test that simultaneously detects antibodies for syphilis and human immunodeficiency virus (HIV).

British Columbia Centre for Disease Control (BCCDC): a public health center focused on communicable disease prevention in British Columbia.

Canadian Paediatric Surveillance Program (CPSP): a program monitoring pediatric conditions, including congenital syphilis, in Canada.

Chembio DPP^®^ Syphilis Screen & Confirm: a rapid diagnostic test that detects antibodies to both treponemal and non-treponemal antigens.

Congenital syphilis: a type of syphilis infection caused by the vertical transmission of a syphilis infection from parent to child during pregnancy, or less commonly, through contact with syphilis lesions at the time of birth.

H.E.R. Pregnancy Program: the Healthy, Empowered, Resilient Pregnancy program, which supports pregnant individuals experiencing conditions of vulnerability by providing culturally-safe and trauma-informed care.

Integrated STBBI strategy: a Canadian approach to addressing sexually transmitted and blood-borne infections by considering the interconnected risk factors and transmission routes, while incorporating disease-specific approaches when necessary.

MedMira Multiplo Rapid TP/HIV test: a rapid point-of-care test that simultaneously detects antibodies for syphilis and human immunodeficiency virus (HIV).

MedMira Reveal Rapid™ TP Test: a rapid diagnostic test used for the detection of syphilis.

National Advisory Committee on STBBI (NAC-STBBI): a Canadian advisory committee on sexually transmitted and blood-borne infections.

Partner Notification Nurse (PNN): a health care professional who ensures contact tracing and partner treatment for STBBIs.

Point-of-care tests (POCT): diagnostic tests performed at or near the site of patient care.

Prenatal syphilis: a syphilis infection present during pregnancy.

Public Health Agency of Canada (PHAC): the federal agency responsible for protecting against threats to public health, preventing and reducing diseases and injury, and promoting health, well-being and equity in Canada.

Sex- and gender-based analysis plus (SGBA Plus): an intersectional analytical approach used to advance equity, diversity and inclusion by assessing how sex, gender, and other diverse identity factors affect how people experience research, policies, programs, and services.

Sexually transmitted and blood-borne infections (STBBIs): infections transmitted sexually or through blood, including but not limited to syphilis, chlamydia, gonorrhea, hepatitis, and human immunodeficiency virus (HIV).

Vertical transmission: the transmission of an infection from a parent to a child during pregnancy or childbirth.

Wellness Wheel Clinic: a non-profit organization that provides accessible and community-focused care to rural and Indigenous communities in Canada.
