# Supplementary material for: The rise of congenital syphilis in Canada: threats and opportunities
Source: Front Public Health. 2025 Jan 22;12:1522698. doi: 10.3389/fpubh.2024.1522698 (PMC11794269; doi:10.3389/fpubh.2024.1522698)
Supplement: Supplementary Table S1 — Literature search inclusion and exclusion criteria. [file Table_1.docx]

Table S1: Literature search inclusion and exclusion criteria.

| Inclusion Criteria | Exclusion Criteria |
| --- | --- |
| Discussed the structural or social determinants of health, health equity factors, or other risk factors associated with congenital syphilis | Did not meet the inclusion criteria |
| Based on the Canadian population | Focused solely on the clinical aspects of congenital syphilis (e.g., signs and symptoms) |
| Published in English or French, only | Older versions of publications by the same author(s) exploring the same topic |
| Published after January 2013 | Reviews that did not contribute additional evidence (e.g., meta-analyses) to that previously reported by the original studies |
| Scientific peer-reviewed articles, governmental reports, conference and article abstracts, and scientific posters | Poster or conference publications that did not provide sufficient information |
|  | Duplicate publications |
| Notes | |
| This search was conducted by two independent reviewers, in collaboration with a Public Health Agency of Canada librarian, using Medline, Embase and Scopus databases. The search was completed on September 8, 2023, and was limited to articles published between 2013 and 2023 in Canada. The main search terms were built around the following concepts: syphilis (infectious syphilis and early congenital syphilis); Canada (all provinces and territories); structural and social determinants of health; health equity factors; and syphilis trends over time, by province and territory. The search was conducted using English terms, but the articles reviewed included those published in both English and French. Identical search strategies were employed to search the grey literature. Various search engines and websites were utilized, including Canadian provincial and territorial websites, as well as the websites of CADTH Grey Matters, the Public Health Agency of Canada, the Canadian Public Health Association and the Canadian Journal of Public Health. Searches were also conducted using the Google Scholar search engine. Only the first five pages of results were considered for each search string. Additional articles were identified by manually scanning the reference lists of retrieved articles and through peer recommendations.  Three rounds of screening were conducted to select relevant studies for this literature review. Title and abstract screenings were conducted to assess if publications met inclusion criteria. Publications related to congenital syphilis were identified during the abstract screening. The publications meeting the inclusion criteria were then selected for a final, full-text screening. Both abstract and full-text screenings were undertaken by two reviewers to help minimize the risk of bias. Any discrepancies were resolved through consensus between the reviewers. From the final selection, the following data items were extracted and saved in an Excel worksheet for narrative synthesis: author, title, year of publication, study design and sample size, objective/research question, location (country/city), province/territory if Canada, study population, priority or key populations disproportionately affected, social/structural determinants of health factors , health equity factors, other risk factors, discussion of factors and explanatory mechanisms and study limitations. There were 25 publications from Canada, with the majority originating from Alberta (n=9), followed by Manitoba (n=5), British Columbia (n=3), Quebec (n=2) and Saskatchewan (n=1). In addition to studies focused on specific provinces and territories, one national study, a Canadian Paediatric Surveillance Program congenital syphilis research project co-led by the Canadian Paediatric Surveillance Program and the Sexually Transmitted and Blood-Borne Infection Surveillance Division of the Public Health Agency of Canada, was included in the review. In this prospective study, cases of congenital syphilis were voluntarily reported by paediatricians between June 2021 and May 2023. The latest published preliminary results (analyzing cases reported until February 2023) include 199 cases of congenital syphilis reported from seven provinces and territories, namely: British Columbia, Alberta, Saskatchewan, Manitoba, Ontario, Newfoundland and Labrador and the Northwest Territories.  The set of selected Canadian publications used data from 2002 to 2023 for their analyses, but slightly less than half (n=12) analyzed data from 2017 onwards. All studies were observational. Most studies were case studies (n=7), retrospective cohort studies (n=6) and case series (n=2). The majority conducted only descriptive analyses. | |
