# Supplementary figures and images for: The rise of congenital syphilis in Canada: threats and opportunities
Source: Front Public Health. 2025 Jan 22;12:1522698. doi: 10.3389/fpubh.2024.1522698 (PMC11794269; doi:10.3389/fpubh.2024.1522698)

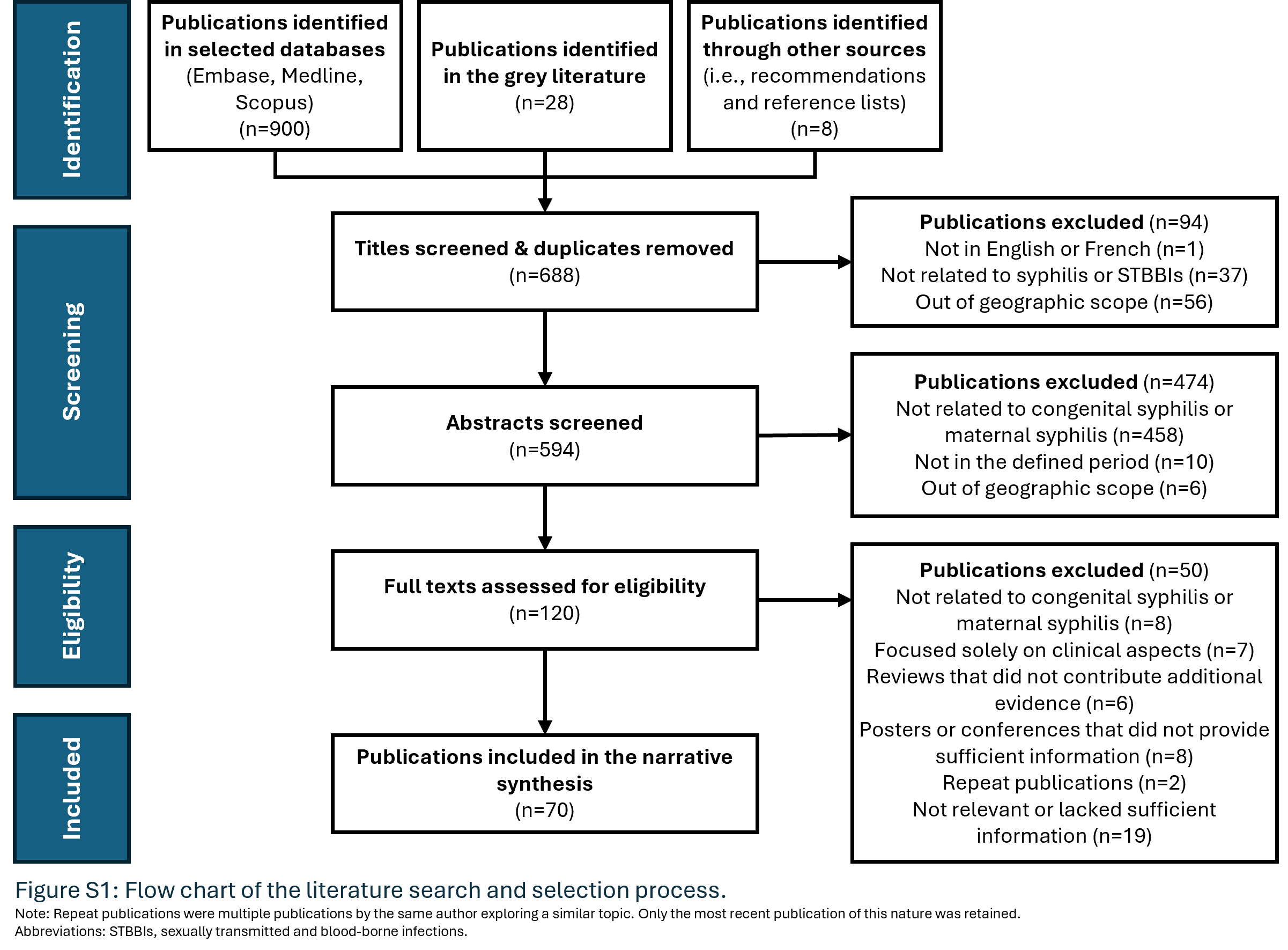

Supplement: Supplementary Figure S1 — Flow chart of the literature search and selection process. [file Image_1.tif]
