# Supplementary material for: The rise of congenital syphilis in Canada: threats and opportunities
Source: Front Public Health. 2025 Jan 22;12:1522698. doi: 10.3389/fpubh.2024.1522698 (PMC11794269; doi:10.3389/fpubh.2024.1522698)
Supplement: Supplementary French abstract S1 — La hausse de la syphilis congénitale au Canada: menaces et opportunités. [file Data_Sheet_1.docx]

# La hausse de la syphilis congénitale au Canada : menaces et opportunités

## Résumé

**Introduction :** Au Canada, les taux de syphilis congénitale ont augmenté rapidement ces dernières années, à la suite d'une augmentation de la syphilis infectieuse. Ces tendances appellent à un examen plus approfondi des opportunités manquées en matière de dépistage, de diagnostic, de traitement et de suivi des personnes enceintes. La situation épidémiologique est d'autant plus grave qu'il existe un traitement efficace de la syphilis pendant la grossesse et que la syphilis congénitale est une issue évitable qui engendre des conséquences négatives à la naissance, telles qu'une fausse couche, une mortinaissance et un décès néonatal, ainsi que des séquelles oculaires, neurologiques, hépatospléniques et musculo-squelettiques qui peuvent durer toute la vie. L'objectif de cette étude est d'examiner les facteurs associés aux tendances de la syphilis congénitale et de mettre en évidence les initiatives et les programmes prometteurs qui, dans tout le pays, s'efforcent de remédier à ces tendances.

**Méthodes :** Une analyse de la littérature, axée sur les études canadiennes, a été réalisée afin d'identifier les facteurs susceptibles d'expliquer l'augmentation continue des taux de syphilis congénitale précoce au cours de la dernière décennie. Une analyse environnementale des initiatives et des programmes fournissant des soins et du soutien aux personnes atteintes de syphilis a également été réalisée.

**Résultats :** Les principaux facteurs identifiés en association avec une issue de syphilis congénitale comprennent l'absence de dépistage prénatal de la syphilis en temps opportun et répété, un traitement prénatal et un suivi inadéquats de l'infection par la syphilis, des obstacles à l'accès aux soins prénatals causés par de multiples déterminants sociaux de la santé qui se recoupent ainsi que par certains déterminants structurels de la santé, et la consommation de substances. Un certain nombre d'initiatives visant à améliorer les soins de la syphilis au sein du système de santé et plusieurs programmes communautaires visant à combler certaines lacunes dans les soins et le soutien aux personnes atteintes de syphilis réalisent des progrès importants dans la lutte contre la situation épidémiologique de la syphilis.

**Discussion :** De nombreux travaux sont en cours à différents niveaux du gouvernement et de la communauté locale pour remédier à la situation. Les principales recommandations visant à maximiser l’impact de la réduction des taux de syphilis infectieuse et de syphilis congénitale comprennent les suivantes : la planification d’une stratégie intégrée pour lutter contre les infections transmissibles sexuellement et par le sang dans leur ensemble ; l’adoption d’une approche plus holistique pour améliorer la santé et le bien-être ; l’élaboration d’interventions ciblées pour s’attaquer aux obstacles structurels et sociaux à l’équité en santé ; et l’adoption d’une approche collaborative de la réponse en impliquant les parties prenantes à plusieurs niveaux, telles que les populations clés, les groupes communautaires, les prestataires de soins de santé et les autorités de santé publique.
